# Selective Grafting of Protease-Resistant Adhesive Peptides on Titanium Surfaces

**DOI:** 10.3390/molecules27248727

**Published:** 2022-12-09

**Authors:** Annj Zamuner, Antonella Pasquato, Ignazio Castagliuolo, Monica Dettin, Paola Brun

**Affiliations:** 1 Department of Civil, Environmental and Architectural Engineering, University of Padua, 35131 Padua, Italy; 2 Department of Industrial Engineering, University of Padua, 35131 Padua, Italy; 3 Department of Molecular Medicine, University of Padua, 35121 Padua, Italy

**Keywords:** titanium, selective grafting, osteoblasts, protease-resistant peptides, retro-inverted peptides, adhesive peptides, vitronectin, bone implants, covalent functionalization, bioactive surface

## Abstract

In orthopedic, dental, and maxillofacial fields, joint prostheses, plates, and screws are widely used in the treatment of problems related to bone tissue. However, the use of these prosthetic systems is not free from complications: the fibrotic encapsulation of endosseous implants often prevents optimal integration of the prostheses with the surrounding bone. To overcome these issues, biomimetic titanium implants have been developed where synthetic peptides have been selectively grafted on titanium surfaces via Schiff base formation. We used the retro-inverted sequence (DHVPX) from [351–359] human Vitronectin and its dimer (D2HVP). Both protease-resistant peptides showed increased human osteoblast adhesion and proliferation, an augmented number of focal adhesions, and cellular spreading with respect to the control. D2HVP-grafted samples significantly enhance Secreted Phosphoprotein 1, Integrin Binding Sialoprotein, and Vitronectin gene expression vs. control. An estimation of peptide surface density was determined by Two-photon microscopy analysis on a silanized glass model surface labeled with a fluorescent analog.

## 1. Introduction

Nowadays, with the increase in life expectancy joined with the reduction in mortality, the elderly population (>65 years old) is expected to double by 2050 in the United States alone [1]. The increase in the percentage of the elderly world population will result in a higher demand for orthopedic and dental implants [2]. For this reason, the interest in finding novel alternative biomaterials for implants able to osseointegrate with the surrounding bone tissue is growing.

Recent studies demonstrated that, in dental application, cemented reconstructions exhibited more serious biological complications (bone loss > 2 mm, implant loss), whilst screw-retained reconstructions exhibited fewer technical and biological complications overall [3,4]. Furthermore, screw-retained reconstructions show apparently higher biological compatibility and therefore seem to be preferable to the cemented ones [3,4].

Titanium and titanium alloys are the gold standard for both dental and orthopedic implants due to their high specific strength (strength per density), high toughness, high corrosion resistance, and biocompatibility [5,6,7]. Unfortunately, although being biocompatible, titanium is not bioactive—that is, such material is not able to drive any specific cellular responses. For this reason, since osseointegration and long-term success of the implant are strictly dependent on the complex interactions occurring at the implant surface/tissue interface, it is important to enrich the implant surface with specific signaling molecules mimicking the extracellular matrix [8]. In turn, these are key factors able to switch cellular responses on and drive cell adhesion, proliferation, and differentiation [9,10,11,12]. To this aim, several methods have been employed to convey specific bioactive motifs on different implant surfaces. The specific covalent grafting is the only one that ensures a stable bond between the motif and the biomaterial, having all peptides oriented in the same way and thus correctly exposing the bioactive sequence to the cells [13,14]. Previously, titanium labeling was achieved using complex techniques consisting of multiple passages based on treatments in organic and acid solutions [15,16,17,18]. In this work, we propose an easier and organic-solvent-free chemical strategy that ensures optimal grafting yields. Moreover, our protocol can be easily translated to a wide variety of substrates, even complex biological matrices.

One of the most extensively studied adhesive sequences is RGD (arginine-glycine-aspartic acid)—a tripeptide pinpointed in the human fibronectin—that interacts with cellular integrins [19,20]. This well-described tripeptide is able to enhance cellular adhesion and proliferation when bonded to the implant surface, but it is not cell-type specific [12,20,21,22,23].

On the other hand, the human vitronectin precursor (HVP) peptide, amino-acids 351–359, is a motif for the heparin binding capability of selectively promoting osteoblasts’ adhesion and adhesion strength in vitro, and enhancing osseointegration in vivo [11,24]. Unfortunately, the HVP peptide is fully cleaved by serum enzymes in only 5 h [16]. Different methods have been investigated to confer stability toward enzyme cleavage, such as the introduction of non-natural amino acids [25] or by making peptide N- and C-termini less accessible to endoproteases (e.g., cyclization) [26]. Among the different strategies so far available, there is the possibility to change all L-amino acids (natural) with the corresponding D enantiomers in the reversed sequence of the original natural peptide. The resulting retro-inverted peptide shows all the sidechains oriented like those of the native peptide. Usually, retro-inverted peptides have a longer in vivo half-life with respect to natural peptides, but the actual bioactivity and cytotoxicity of the retro-inverted sequences must be tested experimentally on a case-by-case basis [27,28].

With the aim to confer stability to the HVP peptide, the retro-inverted analog DHVP and its dimeric sequence D2HVP were designed and synthesized. Moreover, we investigated the ability of DHVP and D2HVP to maintain bioactivities comparable to those of native sequences including the analysis of cytotoxicity and resistance to proteolytic degradation [16,29]. Previous results demonstrated that the dimeric analog D2HVP is able to greatly increase cell adhesion, proliferation, gene expression, and mineralization in vitro when compared both to shorter HVP and DHVP peptides [16,30]: this improved bioactivity of the dimeric peptide in respect to DHVP could be inferred to increased ionic interactions with cellular GAGs or to a greater distance of the bioactive sequence from the implant surface (the first nine amino acids acting as a spacer), resulting in a better exposure of the adhesive sequence to the osteoblasts. To elucidate this point, in this work, a retro-inverted analog of HVP and containing a spacer of the same length as the N-terminal nonapeptide was synthesized and named DHVPX. Furthermore, a control peptide (A-DHVPX) with all Arg replaced by Ala residues was synthesized in order to reduce the pattern of positive charges that is fundamental for initial cellular interactions [11]. The A-DHVPX peptide was further coupled to a fluorescent dye (TAMRA) to allow the peptide surface density quantification using a Two-photon microscope (TPM).

All peptide sequences are reported in Table 1. 

Here, we report and discuss the determination of the surface peptide density able to maximize osteoblast adhesion two hours after seeding and the in vitro biological assays results.

## 2. Results

### 2.1. Surface Peptide Density

Table 2 reports the surface A-DHVPX-TAMRA peptide density measured on glass coverslips. As expected, the superficial density of the peptide is directly proportional to the peptide concentration of the solution used for the surface functionalization. Results demonstrated that by using this covalent anchoring strategy we can easily modulate the degree of functionalization of the glass coverslips. This protocol can be translated to various silanized implant surfaces, such as titanium ones.

### 2.2. Biological Assays

#### 2.2.1. Identification of the Optimal Surface Peptide Density

**Figure 1:**
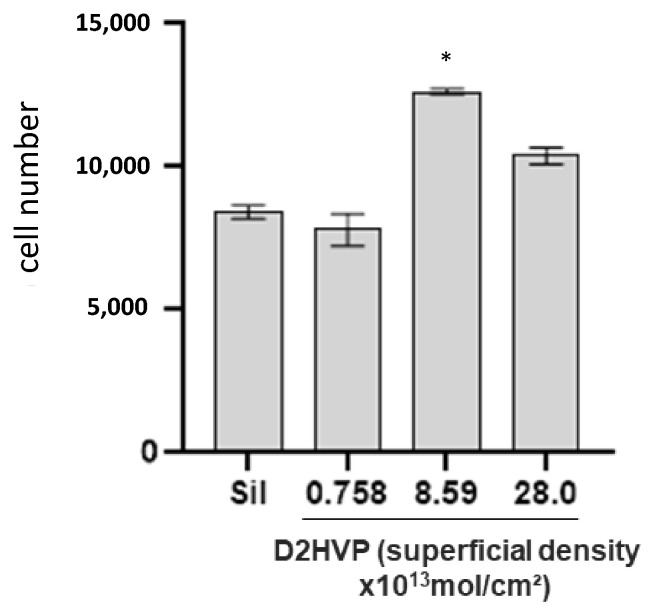
Evaluation of the superficial peptide density for h-osteoblast adhesion maximization. Adhesion assay of human osteoblasts following 2 h incubation on Ti disks grafted with D2HVP at different superficial densities. “Sil” represents the silanized titanium disks. * Denotes *p* < 0.05 vs. Sil.

To identify the most performing surface peptide density in terms of h-osteoblast cell adhesion, we performed preliminary assays to evaluate adhesion of cells following 2 h (h) of incubation. All titanium disks were functionalized with D2HVP at different working concentrations 1 nM, 1 μM, and 1 mM (Section 4.4). As reported in Figure 1, the surface peptide density of 8.59 × 10^−13^ mol/cm^2^, obtained with 1 μM functionalization concentration of D2HVP, gave a significant increase in osteoblast adhesion following 2 h incubation as compared with silanized titanium disks (Sil).

Considering 8.59 × 10^−13^ mol/cm^2^ as the best-performing surface peptide density, all the following bioassays were performed on samples (glass coverslips or titanium disks) functionalized with 1 μM peptide concentration.

#### 2.2.2. Osteoblast Adhesion and Proliferation

**Figure 2:**
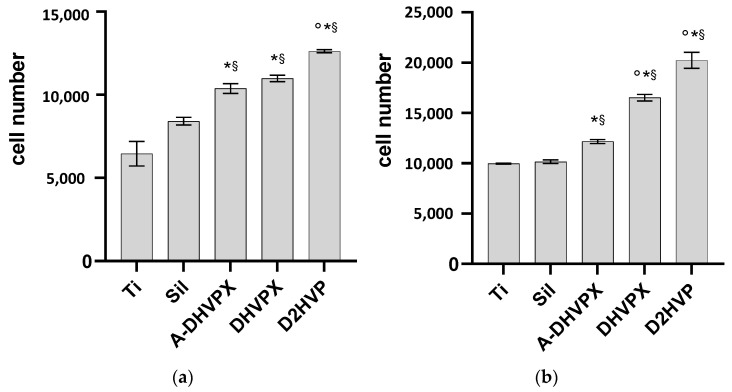
Adhesion and proliferation assays. Adhesion of h-osteoblast cells was assessed at 2 h incubation (**a**) whereas cell proliferation was measured following 24 h incubation (**b**) on differently functionalized Ti disks. “Ti” represents sandblasted and oxidized titanium disks. “Sil” represents the silanized titanium disks. * Denotes *p* < 0.05 vs. Sil; § *p* < 0.05 vs. Ti; ° *p* < 0.05 vs. A-DHVP.

Following 2 h incubation, the titanium disks functionalized with either DHVPX or D2HVP showed a significantly enhanced osteoblast adhesion, with the best result obtained with D2HVP peptide (Figure 2a).

As reported in Figure 2b, all functionalized disks showed a statistically significant improvement in cell adhesion following 24 h culture when compared both to silanized (Sil) and not silanized Ti disks (Ti). Furthermore, DHVPX and D2HVP functionalized samples promoted a significant increase in cell proliferation with respect to the control peptide A-DHVPX. The best result in terms of osteoblast proliferation was achieved with Ti grafted with D2HVP, which gave >100% increase in comparison to the silanized control (Sil).

#### 2.2.3. Immunohistochemistry

**Figure 3:**
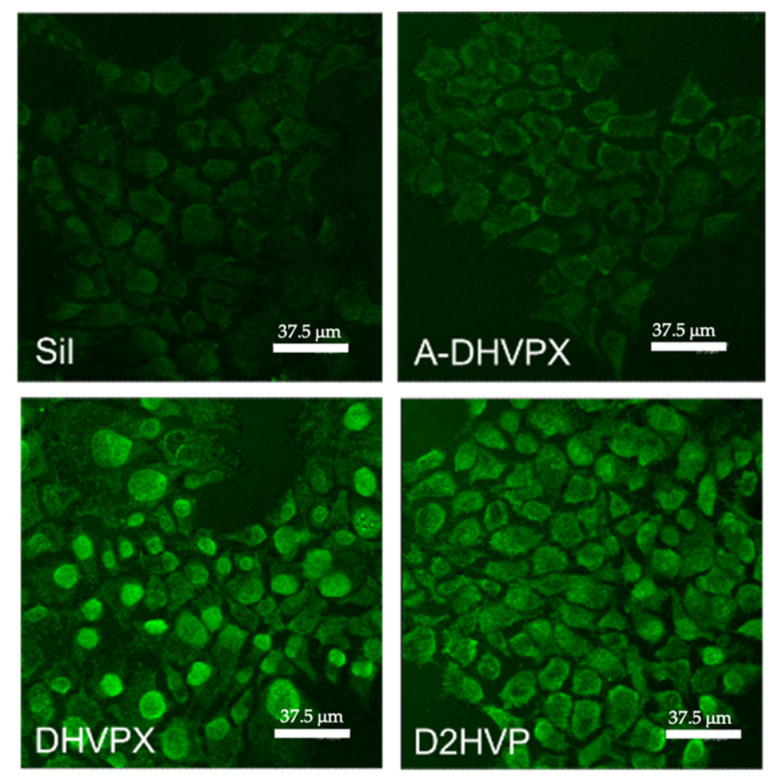
Phosphorylation of FAK. p-FAK detection was detected in h-osteoblast cells by Immunohistochemistry. Cells were cultured on differently functionalized glass coverslips for 24 h. “Sil” represents the silanized glass coverslips. Images were evaluated by confocal microscopy. Scale bars are 37.5 μm.

Focal adhesion kinase (FAK) is a cytoplasmic tyrosine kinase that plays critical roles in integrin-mediated cell adhesion and signal transductions [31]. As reported in Figure 3, peptide functionalization generally induced phosphorylation of FAK (p-FAK) when compared with the naked silanized control (Sil), in line with cell adhesion findings (Figure 2b). FAK phosphorylation was particularly evident in osteoblasts cultured on surfaces treated with DHVPX or D2HVP. In both cases, cells showed homogeneous adhesion zones in which increased labeled areas are present, suggesting the development of focal adhesions attachment with the underlying substrate. 

Regarding osteoblasts morphology, it is evident that on the surfaces treated with the peptides, cells appeared less rounded and with a more articulated form when compared to those seeded on the silanized control (Sil).

#### 2.2.4. Mineralization and Osteogenic Differentiation

**Figure 4:**
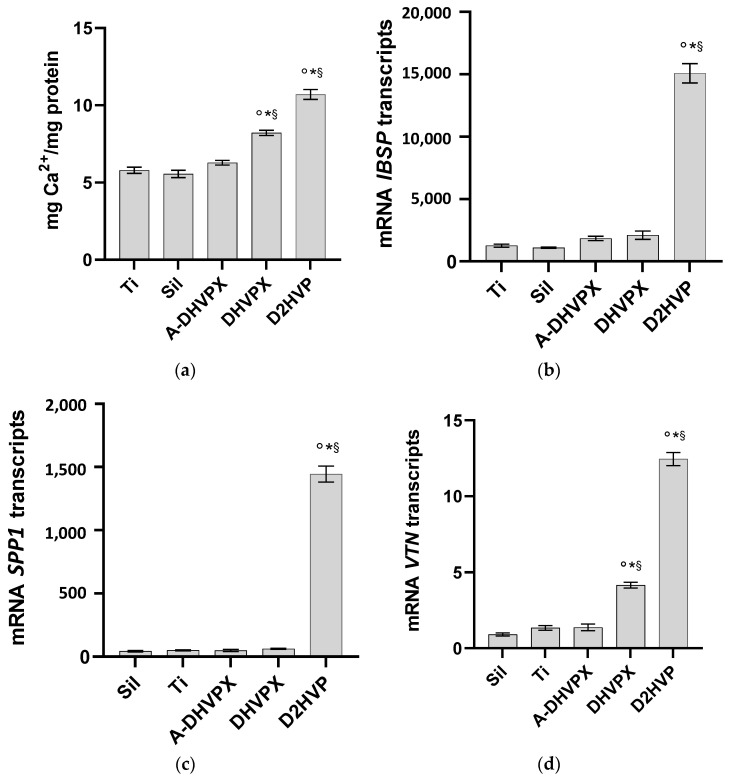
Mineralization and osteogenic differentiation. Osteoblast calcium deposition was evaluated following 7 days of culture (**a**). Gene expression for integrin-binding sialoprotein (IBSP) (**b**), secreted phosphoprotein 1 (SPP1) (**c**), and vitronectin (VTN) (**d**) was evaluated on cells cultured on differently functionalized Ti disks. “Ti” represents sandblasted and oxidized titanium disks. “Sil” represents the silanized titanium disks. * Denotes *p* < 0.05 vs. Sil; § *p* < 0.05 vs. Ti; ° *p* < 0.05 vs. A-DHVP.

A hallmark of osteoblast differentiation and proliferation is the formation and deposition of calcium phosphate crystals. As previously reported, calcium content is undetectable at 2 days of culture, peaks at 7 days, and decreases at 14 days [11]. Therefore, in this study, calcium levels were assessed in h-osteoblasts cultured on differently functionalized glass coverslips for 7 days. Looking at osteoblasts’ calcium deposition (Figure 4a), all functionalized titanium disks showed significantly increased levels as compared with Sil or Ti samples.

In particular, both DHVPX and D2HVP peptides induced a statistically significant increase in calcium deposition when compared to the controls, with the best results recorded by D2HVP, which improved calcium levels by 30% compared to DHVPX. Overall significant performances vs. Sil were DHVPX 48% and D2HVP 93% increases. 

To further evaluate the role of the different functionalization in sustaining human osteoblast differentiation, the expression of genes involved in bone formation was evaluated at 48 h post seeding by quantitative RT-PCR. As reported in Figure 4b,c, D2HVP increased both mRNA-specific transcript levels coding for integrin-binding sialoprotein (IBSP)—a major structural protein of the bone matrix involved in cell attachment [32]—and secreted phosphoprotein 1 (SPP1)—involved in bone mineralization and remodelling. Vitronectin (VTN) mRNA levels were statistically higher both in cells cultured on D2HVP and DHVPX functionalized samples (Figure 4d). In contrast, IBSP, SPP1, and VTN mRNA-specific transcripts did not increase in cells cultured on A-DHVPX functionalized samples compared to silanized titanium (Sil) (Figure 4b–d).

## 3. Discussion

Nowadays the constant request for implants for orthopedics and dentistry applications drives the research toward the development of innovative biomimetic titanium surfaces capable of establishing favorable osteoblast interactions and promoting osteointegration [33,34,35]. Biochemical functionalization of implant surfaces with molecules for specific cell responses is a common approach described in literature. The strategy proposed here consists of the formation of a covalent and selective bond between the silanized implant surface and the biomolecule in order to obtain a peptide orientation favorable for cell interaction. In particular, the proposed surface modification chemistry follows simple protocols which fully avoid the use of organic solvents such as DMF and the strong acids often needed in this type of immobilization [14,16]. In turn, our anchoring strategy is more versatile and could be applied to biological matrices as well [36]. For surface functionalization, we employed aqueous solutions of peptides containing an aldehyde at the C-terminus. These chemical moieties react selectively with the amino groups of the silanized surfaces, with consequent formation of Schiff bases then reduced with NaBCNH_3_. Thanks to the TPM analyses of A-DHVPX-TAMRA functionalized glass surfaces, it was possible to observe how we were able to finely tune the surface density of the anchored biomolecules by simply varying the concentration of the peptide solution used in the functionalization treatment. The A-DHVPX surface densities obtained with peptide solutions of 1 nM, 1 μM, and 1 mM were 0.0758 pmol/cm^2^, 0.859 pmol/cm^2^, and 2.80 pmol/cm^2^, respectively. These results are comparable with surface peptide densities reported in the work of Brun P. et al. [11]. In that paper, a different method was performed for the specific covalent anchoring to silanized titanium disk via sidechain protected peptides. Surface peptide density was determined through radiolabeling and Xray Photoelectron Spectroscopy measurements. In this study, the unspecific peptide binding was not evaluated. Nevertheless, as reported in [37], cells can discriminate between adsorbed biomolecules and covalently anchored ones through their mechanosensory system and, digging into the physisorbed protein layer, cells reach the submerged covalently bonded adhesion motifs in a way to establish a firmer adhesive structure and build thicker stress fibers that improve the mechanical stability of the cytoskeleton. Furthermore, another study [38] demonstrated that an RGD peptide simply mixed into poly-ε-caprolactone was released to around 40% of its total amount already in the first 24 h and, in addition, there was no significant improvement in human umbilical vein endothelial cells adhesion at 24 h from the seeding, unlike the covalently grafted RGD.

In the previous study of Zamuner et al. [16], the h-osteoblast response to DHVP peptides and its dimeric analog D2HVP was compared to HVP (natural sequence) and its dimer (2HVP). The DHVP and D2HVP showed considerable differences in terms of bioactivity. Only D2HVP possessed remarkable properties regarding the (i) promotion of cell adhesion; (ii) formation of membrane protrusions similar to filopodia involved in cell migration, and (iii) expression of important genes related to osteogenesis process (bone sialoprotein, osteopontin, and vitronectin) [16]. These results could be explained by a greater affinity of D2HVP for the membrane cellular GAGs compared to the nonamer DHVP. As an alternative, the terminal portion of D2HVP may be better exposed due to the presence of the C-terminal nine amino acids, which would act as a spacer between the surface and the cells. To test these hypotheses, an additional peptide (DHVPX) carrying a spacer of a suitable length was synthesized. The two peptides were used to functionalize titanium and glass surfaces, whose bioactivity was subsequently evaluated in vitro. 

Results showed that both DHVPX and D2HVP significantly increased osteoblast adhesion at 2 h (*p* < 0.05) compared to both controls, i.e., sandblasted titanium etched with an acid solution (Ti) and silanized titanium (Sil). This positive trend is maintained upon 24 h incubation. At both time points, the D2HVP peptide statistically promotes osteoblast adhesion and proliferation with respect to the control peptides (A-DHVPX), which lack the complete pattern of charges responsible for heparin interactions [39]. On the other hand, DHVPX showed a statistically significant increase in osteoblast proliferation only at 24 h incubation when compared to the control peptide. Intriguingly, MTT tests suggested that the control peptide enhanced cell viability as well when compared to the two control surfaces (Ti and Sil) at both time points. Such finding may be explained taking into consideration the residual positive charges on A-DHVPX which, albeit moderately, can stimulate adhesion mediated by proteoglycans. Concluding, D2HVP induced a higher osteoblast adhesion and proliferation than DHVPX, demonstrating that the C-terminal nonapeptide of D2HVP does not exclusively have the role of a spacer.

Morphological analyses also revealed the ability of both peptides D2HVP and DHVPX to stimulate the development of focal contacts. In fact, the acquisition of fluorescence images in particular revealed more intense discrete areas attributable to the development of focal adhesions with respect to the silanized and A-DHVPX controls. In addition, both peptides promoted cellular spreading more than the control (Sil). Furthermore, both DHVPX and D2HVP peptides showed a significant improvement in calcium deposition with respect to all controls. 

Gene expression evaluation was performed to quantify the levels of mRNA encoding bone sialoprotein (IBSP), osteopontin (SPP1), and vitronectin (VTN) at 48 h from cell seeding on biomimetic titanium disks. Only those functionalized with D2HVP dramatically increased the expression of sialoprotein and osteopontin when compared to all controls. Moreover, VTN gene expression was significantly promoted with respect to the controls by both D2HVP and DHVPX, even if in the first case the expression is considerably greater than in the second one. In line with [16], the D2HVP peptide increased gene expression in a significant way compared to the controls. 

## 4. Materials and Methods

### 4.1. Materials

The solid support resin used for the solid phase peptide synthesis (SPPS) of all peptides was H-Phe-H NovaSyn TG resin from Novabiochem (Merk KGaA, Darmstadt, Germany). All Fmoc-protected **D**-amino acids, 2-(1H-Benzotriazole-1-yl)-1,1,3,3-tetramethyluronium hexafluorophosphate (HBTU), Sodium cyanoborohydride (NaCNBH_3_) and 5(6)-Carboxytetramethylrhodamine (TAMRA), were purchased from Novabiochem (Merk KGaA, Darmstadt, Germany). *N*-Hydroxybenzotriazole (HOBt) was supplied by Advanced Biotech Italia s.r.l. (Seveso, Italy). The Fmoc-7aminoheptanoic (7) acid was from Bachem AG (Bubendorf, Switzerland). Triethylsilane (TES), (3-Aminopropyl)triethoxysilane (APTES), and Acetic Acid were from Sigma-Aldrich (Merk KGaA, Darmstadt, Germany). Dichloromethane (DCM), *N*, *N*-diisopropylethylamine (DIEA), diethyl ether, *N*-Methyl-2-pyrrolidone (NMP), piperidine, and trifluoroacetic acid (TFA) were purchased from Biosolve B.V. (Valkenswaard, The Netherlands). Acetonitrile and *N*, *N*-dimethylformamide were supplied by VWR International S. a. s. (Fontenay-sous-Bois, France). Methanol was purchased from Scharlab (Sentmenat, Spain).

### 4.2. Peptide Synthesis

#### 4.2.1. DHVPX

The DHVPX (IUPAC name: *retro*-*ent*-HVPX) (sequence: H-Tyr-Gly-Lys-Arg-Asn-Arg-His-Arg-Phe-Gly-(Ahp)_3_-Phe-CHO, where Ahp stays for 7-aminoheptanoic acid; the five C-terminal residues G(Ahp)_3_F act as a spacer between the anchoring group (aldehyde) and the other nine residues) was synthesized on H-Phe-H NovaSyn TG resin (0.2 mmol/g) [40] using Fmoc chemistry by a Syro I synthesizer (Multisyntech, Witten, Germany). The sidechain protecting groups were Trt, His, and Asn; Boc, Lys; Pbf, Arg, and tBu, Tyr. The synthesis was carried out using D-amino acids. Every single coupling was performed with 5 eq of amino acid, 5 eq of HBTU/HOBt, and 10 eq of DIEA for 45 min. All couplings were single except for the third coupling which was double. After Fmoc deprotection of the last inserted amino acid, all side-chain protections were removed with pure TFA for 1 h under stirring, then the resin was cleaved using a solution of acetic acid, water, methanol, and dichloromethane (10:5:21:63) for 1 h under stirring. After cleavage, the resin was filtered, the reaction mixture concentrated, and the crude peptide precipitated with cold ethyl ether. The crude peptide (43.4 mg) was dissolved in eluent A (0.05% TFA in H_2_O MilliQ), and the solution was filtered and loaded on Jupiter C_18_ (5 μm, 300 Å, 10 mm × 250 mm, Phenomenex) and separated in the following conditions: eluent A, 0.05% TFA in MilliQ water; eluent B, 0.05% TFA in CH_3_CN; gradient, from 0% B to 15% B in 2 min, then from 15% B to 25% B in 30 min; flow rate, 4 mL/min; detection at 214 nm. The chromatogram of purified DHVPX was obtained using the following: column, Vydac C_18_ Everest (5 μm, 300 Å, 4.6 mm × 250 mm, Grace Davison Discovery Sciences); injection volume, 100 μL of 1 mg/mL peptide solution; flow rate, 1 mL/min; eluent A, 0.05% TFA in water; eluent B, 0.05% TFA in CH_3_CN; gradient, from 15% B to 30% B in 30 min, detection at 214 nm. The retention time resulted in 15.32 min and the final peptide had a purity grade of 93.5%. The identity of the pure peptide was ascertained by Mass Spectrometry (Figure S1 Supplementary materials); experimental mass: 1804.14 Da, theoretical mass: 1803.20 Da (4800 MALDI-TOF/TOF TM analyzer provided with 4000 Series Explorer TM software, Applied Biosystem/MDS Sciex, Framingham, MA, USA).

#### 4.2.2. A-DHVPX

The A-DHVPX peptide (IUPAC name: [Ala^4,6,8^]-*retro-ent*-HVPX) (sequence: H-Tyr-Gly-Lys-Ala-Asn-Ala-His-Ala-Phe-Gly-(Ahp)_3_-Phe-CHO, where Ahp stays for 7-aminoheptanoic acid) was synthesized as a control peptide in which all the Arginine (basic, charge+) residues of DHVP were substituted with Alanine (hydrophobic, not charged) residues. The synthesis, the final Fmoc deprotection, the side-chain groups’ deprotection, and the cleavage from the resin were performed as for the DHVPX peptide. In this case, only two-thirds of the total amount of peptide was cleaved from the resin and from the sidechain protecting groups. Overall, one-third of the peptide, after the final Fmoc deprotection, was kept protected at the side chains on the solid support to proceed with the labeling with the fluorophore TAMRA to obtain A-DHVPX-TAMRA (Section 4.2.3). The crude A-DHVPX peptide (34.3 mg) was dissolved in eluent A (0.05% TFA in H_2_O MilliQ), filtered, and purified through HPLC at the following conditions: column Jupiter C_18_ (5 μm, 300 Å, 10 mm × 250 mm, Phenomenex); eluent A (0.05% TFA in H_2_O MilliQ) and eluent B (0.05% TFA in CH_3_CN); gradient from 0% B to 10% B in 2 min, then from 10% B to 30% B in 60 min; flow rate, 4 mL/min; detection at 214 nm. The chromatogram of purified A-DHVPX was obtained using the following: column, Vydac C_18_ Everest (5 μm, 300 Å, 4.6 mm × 250 mm, Grace Davison Discovery Sciences); injection volume, 100 μL of 1 mg/mL peptide solution; flow rate, 1 mL/min; eluent A, 0.05% TFA in water; eluent B, 0.05% TFA in CH_3_CN; gradient, from 15% B to 40% B in 25 min, detection at 214 nm. The retention time resulted in 13.99 min and the final peptide had a purity grade higher than 99%. The identity of the pure peptide was ascertained by Mass Spectrometry (Figure S2); experimental mass: 1547.98 Da, theoretical mass: 1545.76 Da (4800 MALDI-TOF/TOF TM analyzer provided with 4000 Series Explorer TM software, Applied Biosystem/MDS Sciex, Framingham, MA, USA).

#### 4.2.3. A-DHVPX-TAMRA

The peptide A-DHVPX-TAMRA (IUPAC name: 5(6)-Carboxytetramethylrhodamine-[Ala^4,6,8^]-*retro*-*ent*-HVPX) (sequence: TAMRA-Tyr-Gly-Lys-Ala-Asn-Ala-His-Ala-Phe-Gly-(Ahp)_3_-Phe-CHO, where Ahp stays for 7-aminoheptanoic acid) was prepared in order to quantify the surface peptide density using a two-photon microscope. The coupling of one-third of the sidechain protected A-DHVPX on the solid support was conducted manually by adding 5 eq of TAMRA, 5 eq of HBTU/HOBt, and 10 eq of DIEA in DMF (2.5 mL total volume). The coupling lasted 45 min under agitation. Eventually, the peptide was deprotected and cleaved from the resin following the same procedure used with the DHVPX peptide (Section 4.2.1). The crude A-DHVPX-TAMRA peptide (20.5 mg) was dissolved in 15% *v*/*v* CH_3_CN in H_2_O MilliQ, filtered, and purified through HPLC at the following conditions: column Jupiter C_18_ (5 μm, 300 Å, 10 mm × 250 mm, Phenomenex); eluent A (0.05% TFA in H_2_O MilliQ) and eluent B (0.05% TFA in CH_3_CN); gradient from 0% B to 16% B in 2 min, then from 16% B to 36% B in 60 min; flow rate, 4 mL/min; detection at 214 nm. The chromatogram of purified A-DHVPX-TAMRA was obtained using the following: column, Vydac C_18_ Everest (5 μm, 300 Å, 4.6 mm × 250 mm, Grace Davison Discovery Sciences); injection volume, 100 μL of 1 mg/mL peptide solution; flow rate, 1 mL/min; eluent A, 0.05% TFA in water; eluent B, 0.05% TFA in CH_3_CN; gradient, from 15% B to 40% B in 25 min, detection at 214 nm. The retention time resulted in 17.87 min and the final peptide was 92.6% pure and contained only the first eluting isomer. The identity of the pure peptide was ascertained by Mass Spectrometry (Figure S3); experimental mass: 1961.35 Da, theoretical mass: 1960.21 Da (4800 MALDI-TOF/TOF TM analyzer provided with 4000 Series Explorer TM software, Applied Biosystem/MDS Sciex, Framingham, MA, USA).

#### 4.2.4. D2HVP

The D2HVP peptide (IUPAC name: *retro*-2HVP peptide)(sequence: H-Tyr-Gly-Lys-Arg-Asn-Arg-His-Arg-Phe-Tyr-Gly-Lys-Arg-Asn-Arg-His-Arg-Phe-Phe-CHO) was synthesized on H-Phe-H NovaSyn TG resin (0.2 mmol/g) [40] using Fmoc chemistry by a Syro I synthesizer (Multisyntech, Witten, Germany). The sidechain protecting groups were Trt, His and Asn; Boc, Lys; Pbf, Arg, and tBu, Tyr. The synthesis was carried out using D-amino acids. Each coupling was single and was performed with 5 eq of amino acid, 5 eq of HBTU/HOBt, and 10 eq of DIEA for 45 min. After Fmoc deprotection of the last inserted amino acid, the cleavage from the resin and the side chain deprotection were carried out as in Section 4.2.1. The crude D2HVP peptide (142.5 mg) was dissolved in eluent A (0.05%TFA in H_2_O MilliQ), filtered, and purified through HPLC at the following conditions: column Jupiter C_18_ (5 μm, 300 Å, 10 mm × 250 mm, Phenomenex); eluent A (0.05% TFA in H_2_O MilliQ) and eluent B (0.05% TFA in CH_3_CN); gradient from 0% B to 14% B in 2 min, then from 14% B to 22% B in 32 min; flow rate, 4 mL/min; detection at 214 nm. The chromatogram of purified D2HVP was obtained using the following: column, Vydac C_18_ Everest (5 μm, 300 Å, 4.6 mm × 250 mm, Grace Davison Discovery Sciences); injection volume, 200 μL of 1 mg/mL peptide solution; flow rate, 1 mL/min; eluent A, 0.05% TFA in water; eluent B, 0.05% TFA in CH_3_CN; gradient, from 10% B to 25% B in 30 min, detection at 214 nm. The retention time resulted in 17.57 min and the final peptide was 98.6% pure. The identity of the pure peptide was ascertained by Mass Spectrometry (Figure S4); experimental mass: 2580.59 Da, theoretical mass: 2579.97 Da (4800 MALDI-TOF/TOF TM analyzer provided with 4000 Series Explorer TM software, Applied Biosystem/MDS Sciex, Framingham, MA, USA).

### 4.3. Glass Surface Covalent Grafting

**Figure 5:**
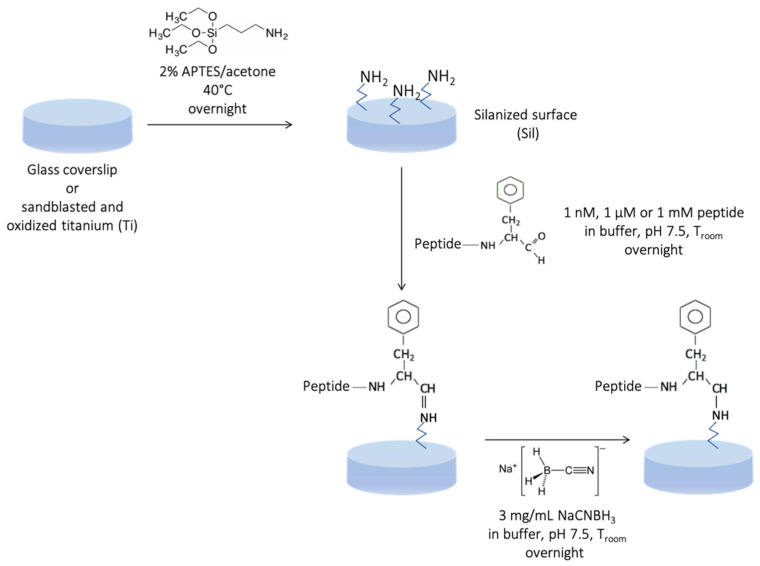
Scheme showing the chemistry of the peptide anchoring strategy onto glass coverslips and titanium surfaces.

Before functionalization, glass coverslips were washed for 3 min in an ultrasound bath with different solvents used in the following order: (i) pure acetone, (ii) 70% *v*/*v* ethanol in H_2_O MilliQ, and (iii) H_2_O MilliQ. The first step involved treatment with 1 N NaOH in H_2_O MilliQ for 1 h at room temperature. Then, the samples were washed three times with H_2_O MilliQ before drying in an oven at 100 °C for 10 min. Coverslips were washed three times with acetone and dried under vacuum for 15 min. Samples were silanized with 2% *v*/*v* APTES in acetone at 40 °C for 24 h. Only silanized glass coverslips were used as control (Sil). After silanization, samples were extensively washed three times with each of these solvents: DCM, acetone, and H_2_O MilliQ. Surface functionalization was carried out using different peptide concentrations: 1 nM, 1 μM, and 1 mM in 20 mM sodium phosphate and 200 mM NaCl buffer at pH 7.5, the reducing agent NaCNBH_3_ was added with a concentration of 3 mg/mL (Figure 5). After functionalization, glass coverslips were washed three times with H_2_O MilliQ and one time with acetonitrile, then samples were dried under vacuum for 1 h at room temperature.

Functionalized glass coverslips were used for surface peptide density quantification with TPM, and for immunohistochemistry assay of human osteoblasts with a confocal microscope.

### 4.4. Titanium Surface Covalent Grafting

Oxidation and silanization of sandblasted Ti (grade 2) disks (Ø 14.2 mm; h 2.5 mm) were performed as reported elsewhere [15]. Only silanized Ti disks were used as control (Sil). The covalent grafting of each peptide was performed as reported in Section 4.3 and is schematized in Figure 5.

### 4.5. Two-Photon Microscope

To quantify the surface peptide density, the fluorescence emitted at 800 nm was measured on glass coverslips functionalized with different A-DHVPX-TAMRA peptide concentrations. A calibration curve was constructed by reading the signals emitted by different A-DHVPX-TAMRA aqueous solutions (10 μL) at concentrations of 1 nM, 100 nM, 1 μM, 10 μM, and 100 μM, respectively (Figure S5). Only one measurement per concentration was performed, considering focal volumes with a square base (ROI) of 484 μm side and with a height (resolution in z) equal to 2 μm. At this point, the fluorescence emitted by glass coverslips functionalized with solutions of A-DHVPX-TAMRA at three different concentrations, 1 nM, 1 μM, and 1 mM was measured in triplicate (for a total of 9 samples). Silanized glass coverslips (3 samples) were used as control. Measurements were conducted on 484 × 484 × 2 μm^3^ focal volume. The average fluorescence intensity of the control samples (Sil) was subtracted from the fluorescence intensity measured for each functionalized glass coverslip. Using the calibration curve, it was then possible to calculate the peptide molar concentration, and consequently the surface peptide density.

### 4.6. Biological Assays

#### 4.6.1. Cell Culture

We obtained human osteoblast (h-osteoblast) cells from explants of cortical mandible bone collected during a surgical procedure from a healthy male subject, 38 years old. The study was approved by the Ethical committee of the University Hospital of Padova (Aut. 4899/AO/20). The patient was informed of the study aims and protocol and gave his written informed consent. Bone fragments were cultured at 37 °C in DMEM supplemented with 20% vol/vol heat-inactivated fetal bovine serum, 10.000 units mL^−1^ of penicillin, 10.000 µg mL^−1^ of streptomycin. We incubated the samples until cells migrated from the bone fragments. At cell confluence, cells were detached using trypsin-EDTA and cultured in DMEM supplemented with 50 mg mL^−1^ ascorbic acid, 10 nM dexametasone, and 10 mM β-glycerophosphate. Osteoblast phenotype was confirmed by the von Kossa staining [41]. Cells were used between passage 4 and passage 7 in culture. 

#### 4.6.2. Cell Adhesion and Proliferation

The h-osteoblast cells were seeded on the functionalized scaffolds (3 × 10^5^ cells) in the complete culture medium. Cell attachment to the scaffolds was assessed by evaluating the viability of cells following 2 h in culture. Thus, cells were rinsed in PBS to remove non-adherent cells and then incubated with MTT (3-(4,5-dimethylthia-zole-2-yl)-2,5-diphenyl tetrazoliumbromide; Merck) solution (5 mg mL^−1^) at 37 °C for 4 h [11]. The reaction was stopped by incubating the cells in 0.01 N HCl—10% *v*/*v* SDS solution for 16 h at room temperature under continuous shaking. The absorbance of the samples was recorded at 620 nm using a microplate reader (Varioskan Lux Reader, Thermo Fisher Scientific, Waltham, MA, USA). 

#### 4.6.3. Immunohistochemistry

The h-osteoblast cells were cultured on functionalized glass coverslips for 24 h at 37 °C. Cells were then washed twice in phosphate buffer saline solution (PBS) and fixed in 4% w/vol buffered PFA for 10 min at room temperature. After extensive washing, cells were permeabilized by incubation with 0.5% vol/vol Triton X-100 in PBS for 15 min at room temperature. Unspecific binding sites were blocked by incubation with 1% w/vol bovine serum albumin (Merck) and then cells were labeled with anti-phospho FAK rabbit monoclonal antibody (clone 31H5L17, Thermo Fischer Scientific) for 2 h. Cells were washed and incubated with the goat anti-rabbit IgG secondary antibody conjugated with Alexa Fluor 488 (Thermo Fischer Scientific) for 45 min at room temperature. The samples were washed three times in PBS and mounted with Prolong Antifade kit (Life Technologies). Samples were imaged using Leica TCSNT/SP2 confocal microscope.

#### 4.6.4. Calcium Deposition

We cultured h-osteoblast cells on functionalized scaffolds for 7 days to assess the calcium content. During the incubation, the culture medium was replaced every 48 h. At the end of incubation, the cell cultures were washed in PBS and incubated for 30 min at 4 °C with 5% w/vol trichloroacetic acid (Merck). The cell extracts (100 μL) were then combined with HCl 3.6 mM, o-cresolphthalein complexone (o-CPC) 100 μM, 2-amino-2methyl-1-propanol 0.142 g/mL (all provided by Merck). A standard curve was obtained by serial dilution (30–0 mg) of CaCO_3_ solution. The absorbance was recorded at 620 nm. Calcium levels were normalized to protein concentration determined in the cell extracts using the bicinchoninic acid method (Pierce, Thermo Fisher Scientific).

#### 4.6.5. Gene Expression

H-osteoblast cells were cultured on different functionalized scaffolds for 48 h at 37 °C. The cells were then subjected to RNA extractionusing the SV Total RNA Isolation System kit (Promega, Milan, Italy). Contaminating DNA was removed by DNase I digestion. Specific mRNA transcript levels coding human integrin binding sialoprotein (IBSP), human secreted phosphoprotein 1 (SPP1), and human vitronectin (VTN) were quantified using the iTaq Universal SYBR Green One-Step Kit (Bio-Rad). The reaction mixture contained 200 nM forward primer, 200 nM reverse primer, iTaq universal SyBR Green reaction mix, iScript reverse transcriptase, and 200 ng total RNA. Real time PCR was performed using ABI PRISM 7700 Sequence Detection System (Applied Biosystems). Human GAPDH was used as the reference gene. Target and reference genes were amplified with efficiencies near 100%. Oligonucleotides used for PCR are listed in Table 3.

#### 4.6.6. Statistical Analysis

For the biological determinations, data are expressed as mean ± standard deviation of at least three independent experiments. Statistical analysis was performed using GraphPad Prism software (GraphPad Software Inc., La Jolla, CA, USA) and statistical significance was calculated using One-way analysis of variance (ANOVA). Differences were considered significant for *p*-value ≤ 0.05.

## 5. Conclusions

In conclusion, the functionalization of titanium surfaces with proteolytically stable bioactive peptides was successfully achieved using a simple chemical strategy. This chemical strategy allowed us to easily modulate the surface peptide density. By similarity, we expect the technique to be suitable for the functionalization of a variety of different implants, even biological matrices. Moreover, the biological assays here carried out pinpointed the dimeric analog D2HVP as the best option for promoting cell adhesion and proliferation, favoring the formation of adhesion focal contacts, and increasing the expression of genes involved in osteogenesis. Eventually, D2HVP is a promising biomolecule for developing prosthetic implants able to drive osseointegration in vivo.

## Figures and Tables

**Table 1:** Peptide sequences.

Peptide	Sequence
HVP	H-Phe-Arg-His-Arg-Asn-Arg-Lys-Gly-Tyr-OH
DHVP	H-D-Tyr-Gly-D-Lys-D-Arg-D-Asn-D-Arg-D-His-D-Arg-D-Phe-OH
DHVPX	H-D-Tyr-Gly-D-Lys-D-Arg-D-Asn-D-Arg-D-His-D-Arg-D-Phe-Gly-Ahp-Ahp-Ahp-Phe-CHO
A-DHVPX	H-D-Tyr-Gly-D-Lys-D-Ala-D-Asn-D-Ala-D-His-D-Ala-D-Phe-Gly-Ahp-Ahp-Ahp-Phe-CHO
A-DHVPX-TAMRA	TAMRA-D-Tyr-Gly-D-Lys-D-Ala-D-Asn-D-Ala-D-His-D-Ala-D-Phe-Gly-Ahp-Ahp-Ahp-Phe-CHO
D2HVP	H-D-Tyr-Gly-D-Lys-D-Arg-D-Asn-D-Arg-D-His-D-Arg-D-Phe-D-Tyr-Gly-D-Lys-D-Arg-D-Asn-D-Arg-D-His-D-Arg-D-Phe-Phe-CHO

HVP is composed by L-amino acids whilst all the other sequences are made of D-Amino acids; sequences are reported using three letter code. Ahp stays for 7-aminoheptanoic acid. TAMRA refers to 5(6)-Carboxytetramethylrhodamine.

**Table 2:** Evaluation of differently functionalized samples at TPM.

[A-DHVPX-TAMRA]	Average Concentration/ Focal Volume [M]	Number of Moles/ Focal Volume	Superficial Peptide Density [mol/cm^2^]
1 nM	3.79 × 10^−7^ ± 2.80 × 10^−7^	1.78 × 10^−16^	7.58 × 10^−14^
1 μM	4.29 × 10^−6^ ± 1.28 × 10^−6^	2.01 × 10^−15^	8.59 × 10^−13^
1 mM	1.40 × 10^−5^ ± 1.04 × 10^−5^	6.56 × 10^−15^	2.80 × 10^−12^

**Table 3:** Oligonucleotide sequences of the genes evaluated by quantitative PCR.

Gene	Sequence
GAPDH	fw 5′-agtgccagcctcgtcccgta-3′ rv 5′-caggcgcccaatacggccaa-3′
IBSP	fw 5′-ttggtttgcacatttaagta-3′ rv 5′-tggaacctgaggctctt-3′
SPP1	fw 5′-cgcagacctgacatccagta-3′ rv 5′-ggctgtcccaatcagaagg-3′
VTN	fw 5′-ggaggacatcttcgagcttct-3′ rv 5′-gctaatgaactggggctgtc-3′

fw: forward; rv: reverse.

## Data Availability

Not applicable.

## Supplementary Materials

The following supporting information can be downloaded at: https://www.mdpi.com/article/10.3390/molecules27248727/s1, Figure S1: Mass Spectra of DHVPX peptide; Figure S2: Mass Spectra of A-DHVPX peptide; Figure S3: Mass Spectra of A-DHVPX-TAMRA peptide; Figure S4: Mass Spectra of D2HVP peptide; Section 1S: TPM Calibration curve; Figure S5: TPM calibration curve.

## Sample Availability

All samples are available from the authors.

## References

[1] Ortman J.M., Velkoff V.A., Hogan H. (2014). An Aging Nation: The Older Population in the United States.

[2] Srinivasan M., Meyer S., Mombelli A., Müller F. (2016). Dental implants in the elderly population: A systematic review and meta-analysis. Clin. Oral Implant. Res..

[3] Sailer I., Mühlemann S., Zwahlen M., Hämmerle C.H.F., Schneider D. (2012). Cemented and screw-retained implant reconstructions: A systematic review of the survival and complication rates. Clin. Oral Implant. Res..

[4] Wittneben J.-G., Millen C., Brägger U. (2014). Clinical performance of screw-versus cement-retained fixed implant-supported reconstructions—A systematic review. Int. J. Oral Maxillofac. Implant..

[5] Goriainov V., Cook R., Latham J.M., Dunlop D.G., Oreffo R.O. (2014). Bone and metal: An orthopaedic perspective on osseointegration of metals. Acta Biomater..

[6] Mantripragada V.P., Lecka-Czernik B., Ebraheim N.A., Jayasuriya A.C. (2012). An overview of recent advances in designing orthopedic and craniofacial implants. J. Biomed. Mater. Res. Part A.

[7] Lewallen E.A., Riester S.M., Bonin C.A., Kremers H.M., Dudakovic A., Kakar S., Cohen R.C., Westendorf J.J., Lewallen D.G., van Wijnen A.J. (2015). Biological strategies for improved osseointegration and osteoinduction of porous metal orthopedic implants. Tissue Eng. Part B Rev..

[8] Chen Q., Zhang D., Gu J., Zhang H., Wu X., Cao C., Zhang X., Liu R. (2021). The impact of antifouling layers in fabricating bioactive surfaces. Acta Biomater..

[9] Mavrogenis A.F., Dimitriou R., Parvizi J., Babis G.C. (2009). Biology of implant osseointegration. J. Musculoskelet. Neuronal Interact..

[10] Smeets R., Stadlinger B., Schwarz F., Beck-Broichsitter B., Jung O., Precht C., Kloss F., Gröbe A., Heiland M., Ebker T. (2016). Impact of dental implant surface modifications on osseointegration. BioMed Res. Int..

[11] Brun P., Scorzeto M., Vassanelli S., Castagliuolo I., Palù G., Ghezzo F., Messina G.M.L., Iucci G., Battaglia V., Sivolella S. (2013). Mechanisms underlying the attachment and spreading of human osteoblasts: From transient interactions to focal adhesions on vitronectin-grafted bioactive surfaces. Acta Biomater..

[12] Sawyer A.A., Hennessy K.M., Bellis S.L. (2007). The effect of adsorbed serum proteins, RGD and proteoglycan-binding peptides on the adhesion of mesenchymal stem cells to hydroxyapatite. Biomaterials.

[13] Jurczak P., Witkowska J., Rodziewicz-Motowidło S., Lach S. (2020). Proteins, peptides and peptidomimetics as active agents in implant surface functionalization. Adv. Colloid Interface Sci..

[14] Dettin M., Herath T., Gambaretto R., Iucci G., Battocchio C., Bagno A., Ghezzo F., Di Bello C., Polzonetti G., Di Silvio L. (2009). Assessment of novel chemical strategies for covalent attachment of adhesive peptides to rough titanium surfaces: XPS analysis and biological evaluation. J. Biomed. Mater. Res. Part A.

[15] Dettin M., Zamuner A., Iucci G., Messina G.M.L., Battocchio C., Picariello G., Gallina G., Marletta G., Castagliuolo I., Brun P. (2014). Driving h-osteoblast adhesion and proliferation on titania: Peptide hydrogels decorated with growth factors and adhesive conjugates. J. Pept. Sci..

[16] Zamuner A., Brun P., Scorzeto M., Sica G., Castagliuolo I., Dettin M. (2017). Smart biomaterials: Surfaces functionalized with proteolytically stable osteoblast-adhesive peptides. Bioact. Mater..

[17] Conde J., Dias J.T., Grazú V., Moros M., Baptista P.V., de la Fuente J.M. (2014). Revisiting 30 years of biofunctionalization and surface chemistry of inorganic nanoparticles for nanomedicine. Front. Chem..

[18] Sapsford K.E., Algar W.R., Berti L., Gemmill K.B., Casey B.J., Oh E., Stewart M.H., Medintz I.L. (2013). Functionalizing nanoparticles with biological molecules: Developing chemistries that facilitate nanotechnology. Chem. Rev..

[19] Yamada K.M. (1991). Adhesive recognition sequences. J. Biol. Chem..

[20] Hersel U., Dahmen C., Kessler H. (2003). RGD modified polymers: Biomaterials for stimulated cell adhesion and beyond. Biomaterials.

[21] Ruoslahti E. (1996). RGD and other recognition sequences for integrins. Annu. Rev. Cell Dev. Biol..

[22] Barczyk M., Carracedo S., Gullberg D. (2010). Integrins. Cell Tissue Res..

[23] Yang M., Zhang Z.-C., Liu Y., Chen Y.-R., Deng R.-H., Zhang Z.-N., Yu J.-K., Yuan F.-Z. (2021). Function and mechanism of RGD in bone and cartilage tissue engineering. Front. Bioeng. Biotechnol..

[24] Cacchioli A., Ravanetti F., Bagno A., Dettin M., Gabbi C. (2009). Human vitronectin-derived peptide covalently grafted onto titanium surface improves osteogenic activity: A pilot in vivo study on rabbits. Tissue Eng. Part A.

[25] Zubrzak P., Williams H., Coast G.M., Isaac R.E., Reyes-Rangel G., Juaristi E., Zabrocki J., Nachman R.J. (2007). β-amino acid analogs of an insect neuropeptide feature potent bioactivity and resistance to peptidase hydrolysis. Biopolymers.

[26] Fetse J., Zhao Z., Liu H., Mamani U.-F., Mustafa B., Adhikary P., Ibrahim M., Liu Y., Patel P., Nakhjiri M. (2022). Discovery of cyclic peptide inhibitors targeting PD-L1 for cancer immunotherapy. J. Med. Chem..

[27] Doti N., Mardirossian M., Sandomenico A., Ruvo M., Caporale A. (2021). Recent applications of retro-inverso peptides. Int. J. Mol. Sci..

[28] Rai J. (2019). Peptide and protein mimetics by retro and retroinverso analogs. Chem. Biol. Drug Des..

[29] Ravanetti F., Gazza F., D’Arrigo D., Graiani G., Zamuner A., Zedda M., Manfredi E., Dettin M., Cacchioli A. (2018). Enhancement of peri-implant bone osteogenic activity induced by a peptidomimetic functionalization of titanium. Ann. Anat. Anat. Anz..

[30] Zamuner A., Brun P., Ciccimarra R., Ravanetti F., Veschini L., Elsayed H., Sivolella S., Iucci G., Porzionato A., Silvio L.D. (2021). Biofunctionalization of bioactive ceramic scaffolds to increase the cell response for bone regeneration. Biomed. Mater..

[31] Zhao X., Guan J.-L. (2011). Focal adhesion kinase and its signaling pathways in cell migration and angiogenesis. Adv. Drug Deliv. Rev..

[32] Fisher L.W., McBride O.W., Termine J.D., Young M.F. (1990). Human bone sialoprotein. Deduced protein sequence and chromosomal localization. J. Biol. Chem..

[33] Griggs J.A. (2017). Dental implants. Dent. Clin. N. Am..

[34] Rupp F., Liang L., Geis-Gerstorfer J., Scheideler L., Hüttig F. (2018). Surface characteristics of dental implants: A review. Dent. Mater..

[35] Yang B.-C., Zhou X.-D., Yu H.-Y., Wu Y., Bao C.-Y., Man Y., Cheng L., Sun Y. (2019). Advances in titanium dental implant surface modification. West China J. Stomatol..

[36] Dal Sasso E., Zamuner A., Filippi A., Romanato F., Palmosi T., Vedovelli L., Gregori D., Gómez Ribelles J.L., Russo T., Gloria A. (2021). Covalent functionalization of decellularized tissues accelerates endothelialization. Bioact. Mater..

[37] Battista E., Causa F., Lettera V., Panzetta V., Guarnieri D., Fusco S., Gentile F., Netti P.A. (2015). Ligand engagement on material surfaces is discriminated by cell mechanosensoring. Biomaterials.

[38] Dettin M., Zamuner A., Roso M., Iucci G., Samouillan V., Danesin R., Modesti M., Conconi M.T. (2015). Facile and selective covalent grafting of an RGD-peptide to electrospun scaffolds improves HUVEC adhesion: Facile and selective covalent grafting. J. Pept. Sci..

[39] Dettin M., Conconi M.T., Gambaretto R., Bagno A., Di Bello C., Menti A.M., Grandi C., Parnigotto P.P. (2005). Effect of synthetic peptides on osteoblast adhesion. Biomaterials.

[40] Ede N.J., Eagle S.N., Wickham G., Bray A.M., Warne B., Shoemaker K., Rosenberg S. (2000). Solid phase synthesis of peptide aldehyde protease inhibitors. Probing the proteolytic sites of hepatitis C virus polyprotein. J. Pept. Sci..

[41] Lee G. (1968). Luna Manual of Histologic Staining Methods of the Armed Forces Institute of Pathology.

