# Supplementary material for: Selective Grafting of Protease-Resistant Adhesive Peptides on Titanium Surfaces
Source: Molecules. 2022 Dec 9;27(24):8727. doi: 10.3390/molecules27248727 (PMC9781125; doi:10.3390/molecules27248727)
Supplement: Supplementary file 1 [file molecules-27-08727-s001.zip › molecules-2029348-supplementary.pdf]

Supplementary Materials

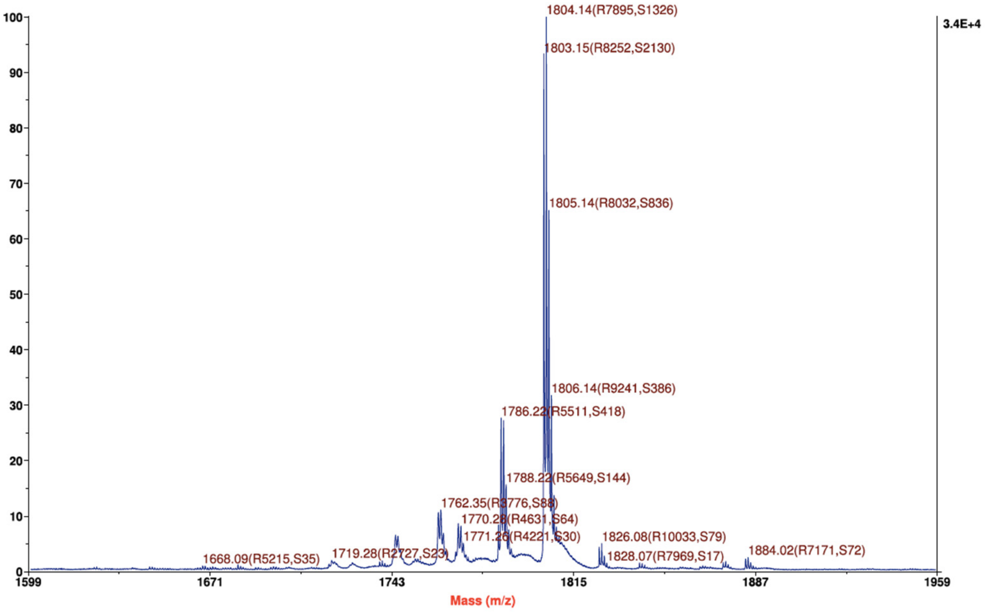

Figure S1. Mass Spectra of DHVPX peptide. The identity of the pure peptide was ascertained by Mass Spectrometry; experimental mass: 1804.14 Da, theoretical mass: 1803.20 Da (4800 MALDI-TOF/TOF TM analyzer provided with 4000 Series Explorer TM software, Applied Biosystem/MDS Sciex, CA, USA).

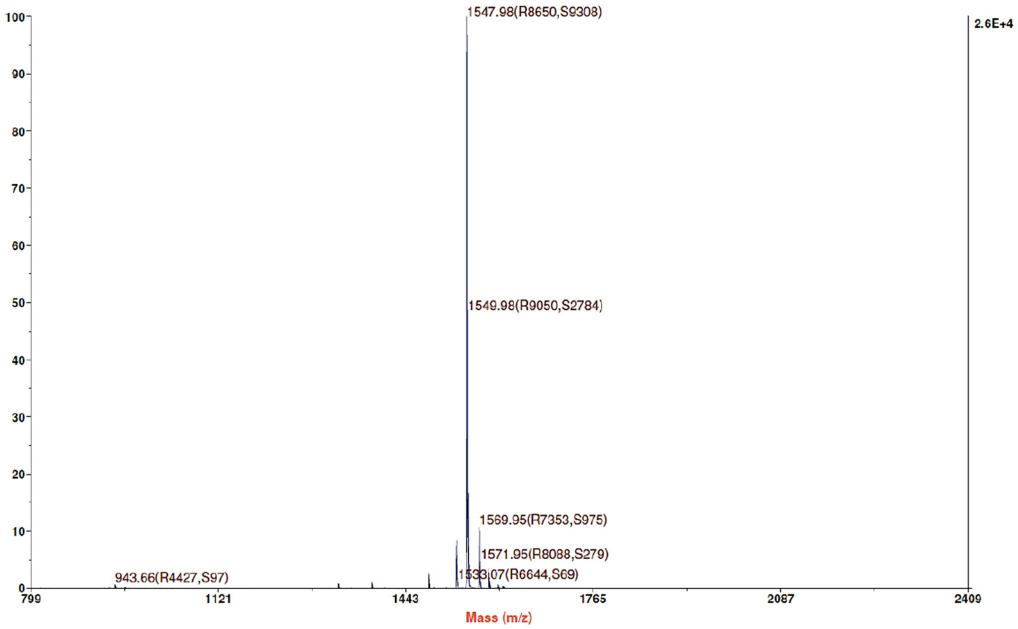

Figure S2. Mass Spectra of A-DHVPX peptide. The identity of the pure peptide was ascertained by Mass Spectrometry; experimental mass: 1547.98 Da, theoretical mass: 1545.76 Da (4800 MALDI-TOF/TOF TM analyzer provided with 4000 Series Explorer TM software, Applied Biosystem/MDS Sciex, CA, USA).

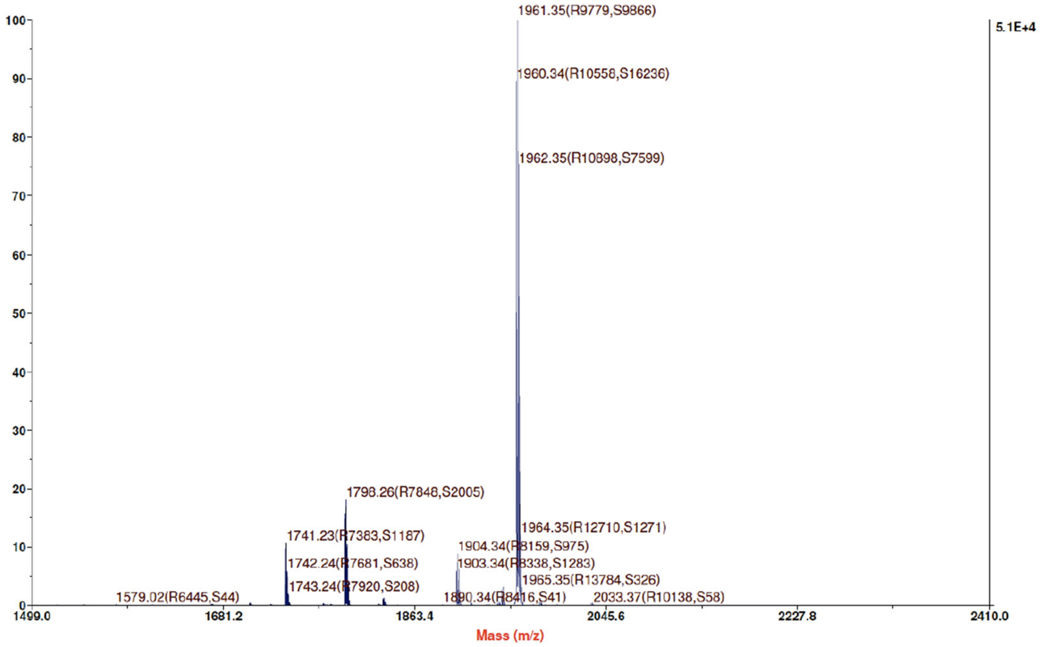

Figure S3. Mass Spectra of A-DHVPX-TAMRA peptide. The identity of the pure peptide was ascertained by Mass Spectrometry; experimental mass: 1961.35 Da, theoretical mass: 1960.21 Da (4800 MALDI-TOF/TOF TM analyzer provided with 4000 Series Explorer TM software, Applied Biosystem/MDS Sciex, CA, USA).

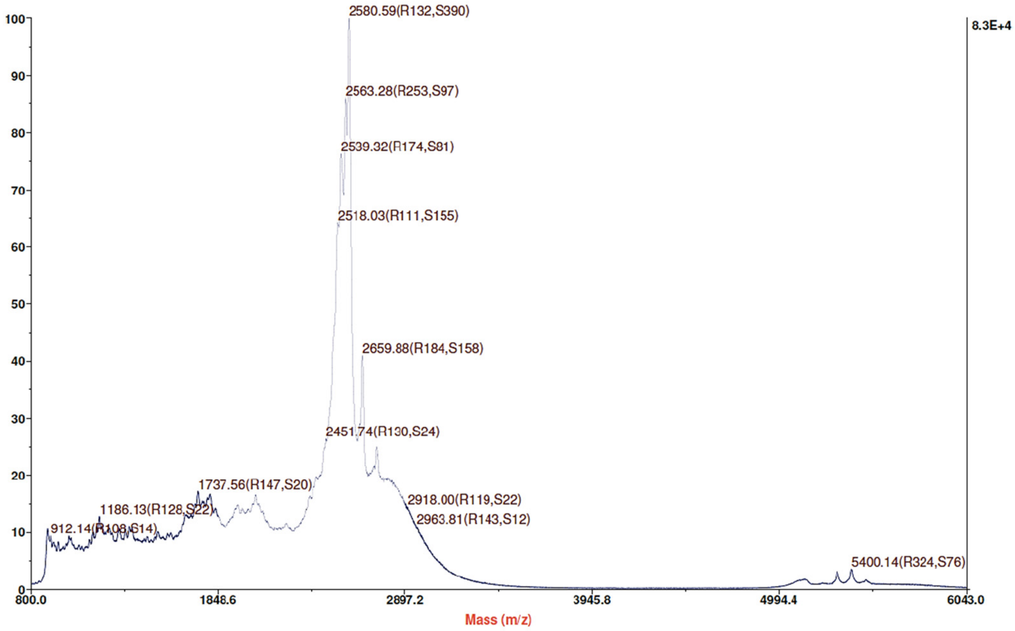

Figure S4. Mass Spectra of D2HVP peptide. The identity of the pure peptide was ascertained by Mass Spectrometry; experimental mass: 2580.59 Da, theoretical mass: 2579.97 Da (4800 MALDI-TOF/TOF TM analyzer provided with 4000 Series Explorer TM software, Applied Biosystem/MDS Sciex, CA, USA).

1S. TPM Calibration curve

A calibration curve was constructed by reading the signals emitted by different A-DHVPX-TAMRA aqueous solutions (10 μL) at concentrations of 1 nM, 100 nM, 1 μM, 10 μM, and 100 μM, respectively and subtracting the background signal of pure H_2O (Figure S5). Only one measurement per concentration was performed, considering focal volumes with a 484 μm square base (ROI) and 2 μm height (resolution in z). Eventually, the interpolation line with its equation has been calculated.

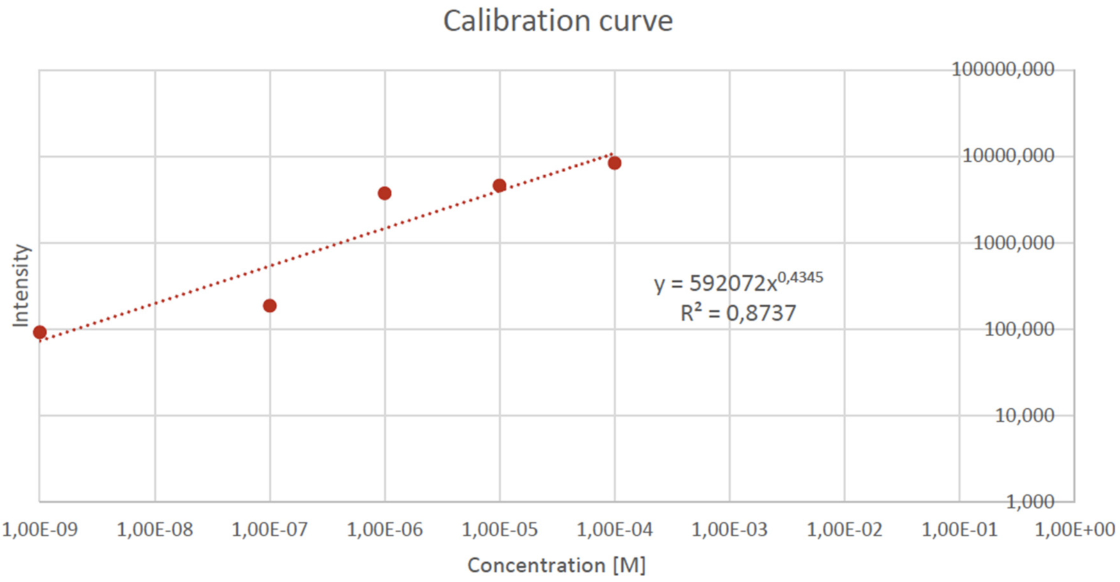

Figure S5. TPM calibration curve.
